# Vascularized solid iris lesion in a 3 year old child: 5 years of follow up

**DOI:** 10.1186/s12886-016-0267-4

**Published:** 2016-06-15

**Authors:** Antonio Maria Fea, Cristina Briamonte, Vittoria Aragno, Federico Maria Grignolo

**Affiliations:** Department of Ophthalmology and Visual Sciences, Ospedale Oftalmico, Via Juvarra 19, Turin, Italy

**Keywords:** Iris lesion, Pediatric age, Anterior segment optical coherence tomography

## Abstract

**Background:**

Iris tumors are rare in young patients. When an iris lesion occurs in a pediatric patient, it can be difficult to classify because of the wide spectrum of iris proliferations.

**Case presentation:**

We report on an unusual case of a vascularized iris lesion in a three year old Caucasian patient, with no symptoms and no visual impairment. We evaluated in a 50-month follow up with non-invasive diagnostic tools in order to avoid eye biopsy.

**Conclusion:**

We focused attention on the differential diagnoses and underlined the role of non-invasive diagnostic tools in a child to avoid or postpone the eye biopsy. We performed a review of the literature to identify the best medical practice in pediatric iris lesions with atypical characteristics.

## Background

In a retrospective case series of 3680 patients, iris tumors predominantly affected Caucasians (96 %) and were rare in patients under 20 years of age (12 %) [1]. There is a wide spectrum of iris tumors and there are few comprehensive series on the full array of clinical ocular manifestations [1]. Morphologic heterogeneity among melanocytic proliferations is a common challenge in the diagnosis of melanoma. In particular, atypical melanocytic lesions in children, adolescents, and young adults may be difficult to classify because of a significant morphologic overlap with melanoma. The clinical differentiation of iris lesions is based upon careful slit lamp examination and gonioscopy and is confirmed by ocular imaging with ultrasound biomicroscopy (UBM) and in some cases, anterior segment optical coherence tomography (AS OCT) [2].

The correct diagnosis can be particularly challenging in younger patients because serial examinations under anesthesia are required to accurately follow the progression of the tumor. We report on a 3 year old child in whom an abrupt onset of an iris mass was noted by his parents over three weeks.

## Case presentation

**Fig. 1:**
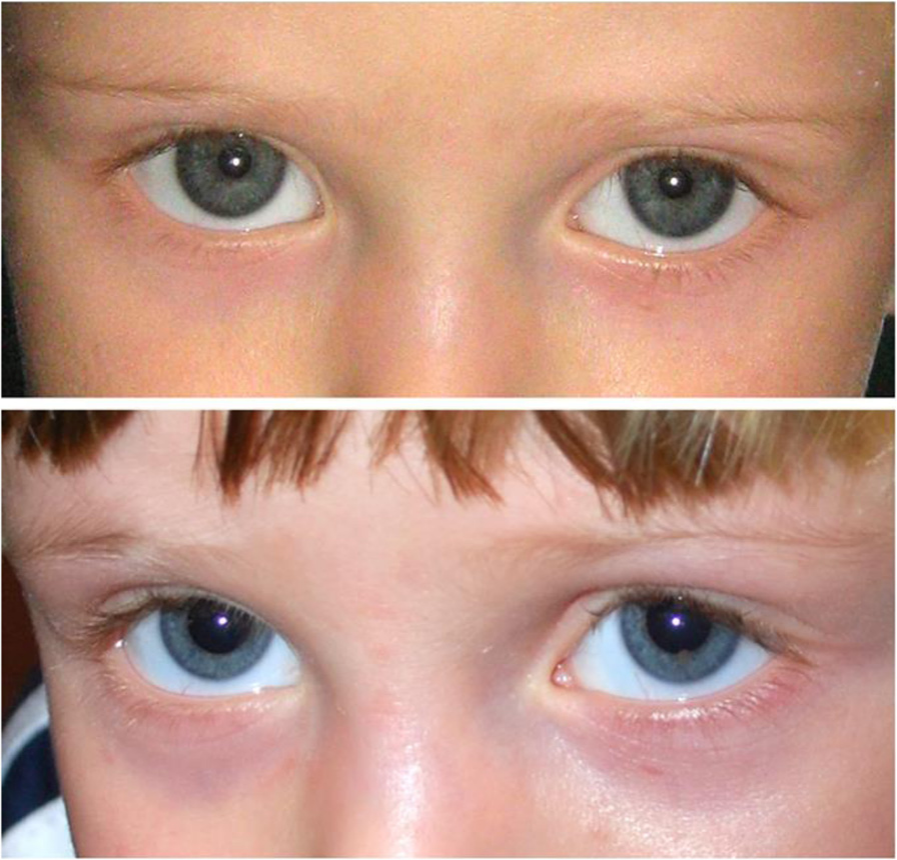
Two consecutive pictures of the patient before and after the first presentation of lesion: The onset of the iris lesion. The two pictures were taken respectively on October 2009 (first row, no evidence of the lesion) and December 2009 (second row, first appearance of the lesion)

A three year-old Caucasian male patient was referred to our clinic in October 2010 for evaluation of a mass on the iris of his right eye. The lesion was originally noted approximately 2 months earlier and the parents observed a progressive increase in its size over the prior three weeks. (Fig. 1)

The parents did not report any subjective complaint of the child. Family and medical history were negative.

The patient was orthophoric with normal binocular visual skills (tracking, convergence, fusion and stereoacuity). His best corrected visual acuity was 10/10 in both eyes (Tumbling E).

The right eye anterior segment examination revealed an elevated, solid, slightly vascularized, amelanotic, light pink mass situated at the 6 clock hour of the iris, extended for 1 clock hour, without satellite lesions. The patient’s anterior left eye segment was clear, without cell and flare, deep, well formed, and did not display iris heterochromia when compared to the fellow eye. Pupils were isocoric and the light reflexes were normal.

Because of the young age of the patient, gonioscopic evaluation of the right eye was not possible.

The patient was referred to Prof Zografos in Lausanne who is one of the European Referring Centers for eye neoplastic diseases and evaluated in January 2011, where he underwent examination under anesthesia. With the aid of operating microscope and a portable slit lamp, they found a vascularized lesion that seemed to originate from the superficial iris stroma. The intraocular pressure was normal in both eyes. The intraoperative gonioscopy revealed a totally open-angle structure without any involvement of the ciliary body.

**Fig. 2:**
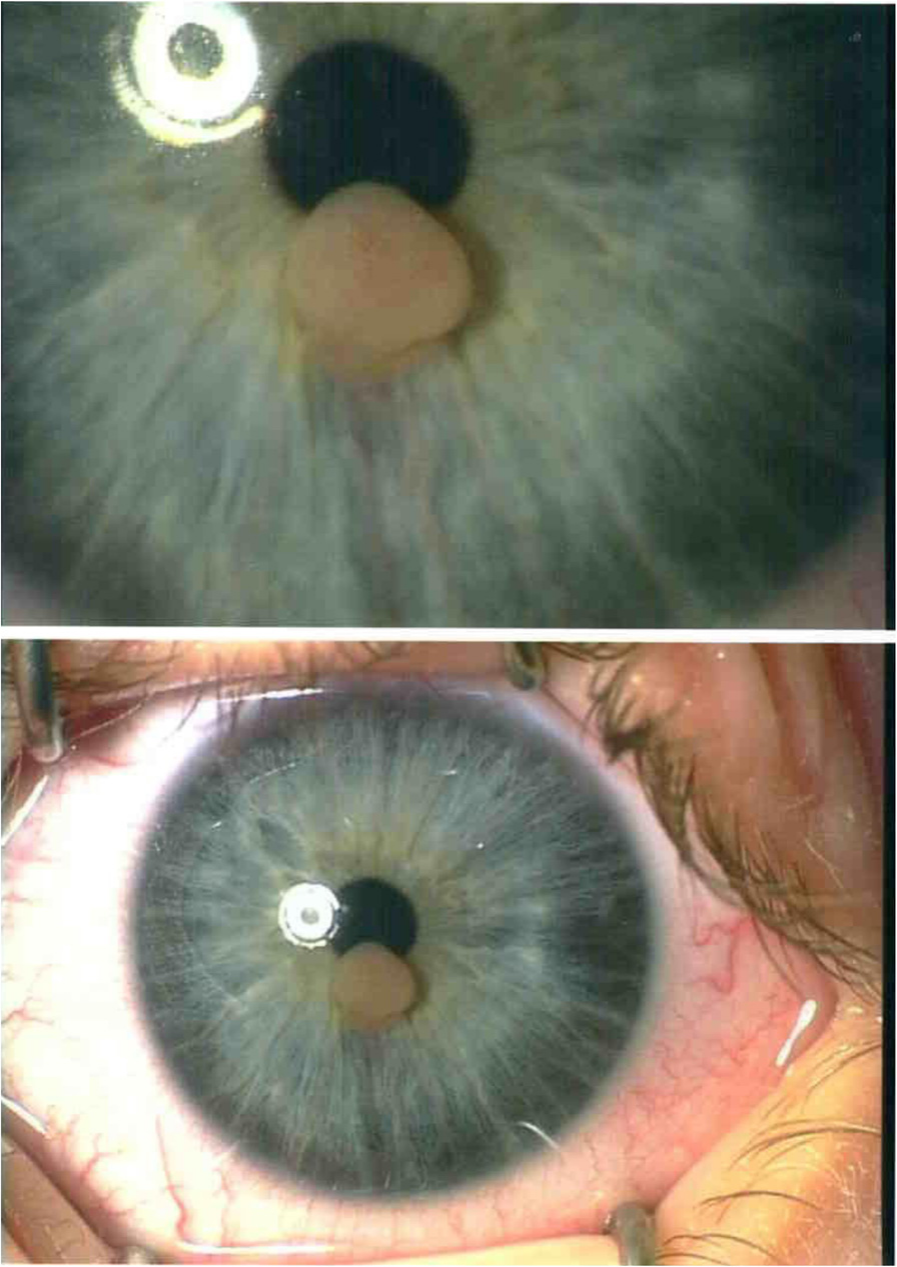
slit lamp anterior segment photography in November 2012: the pictures demonstrates the presence of an iris vascularized pink lesion

**Fig. 3:**
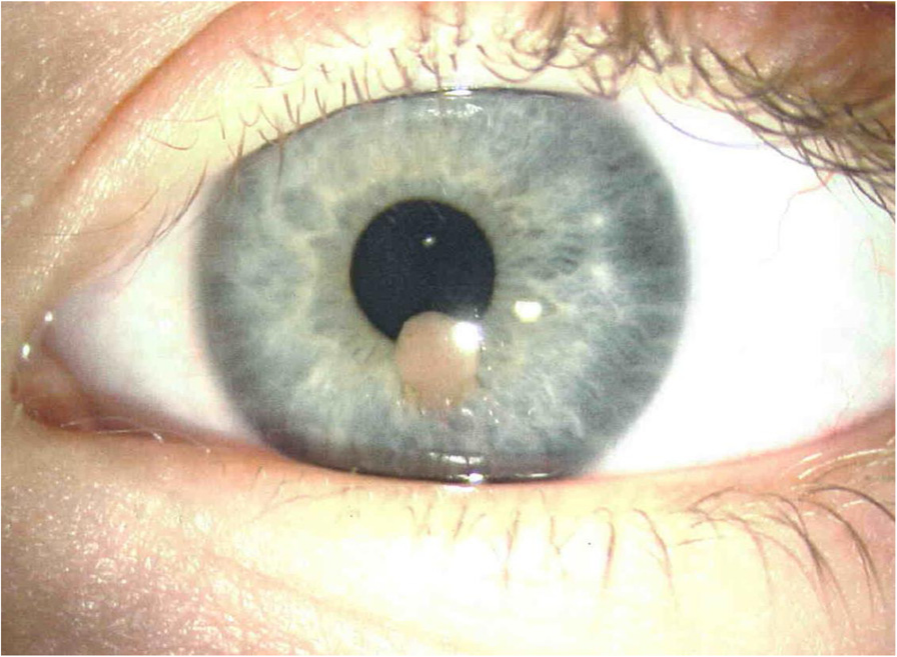
slit lamp anterior segment photography on April 2014: the picture demonstrates the stability of dimension and vascularization throughout months

**Fig. 4:**
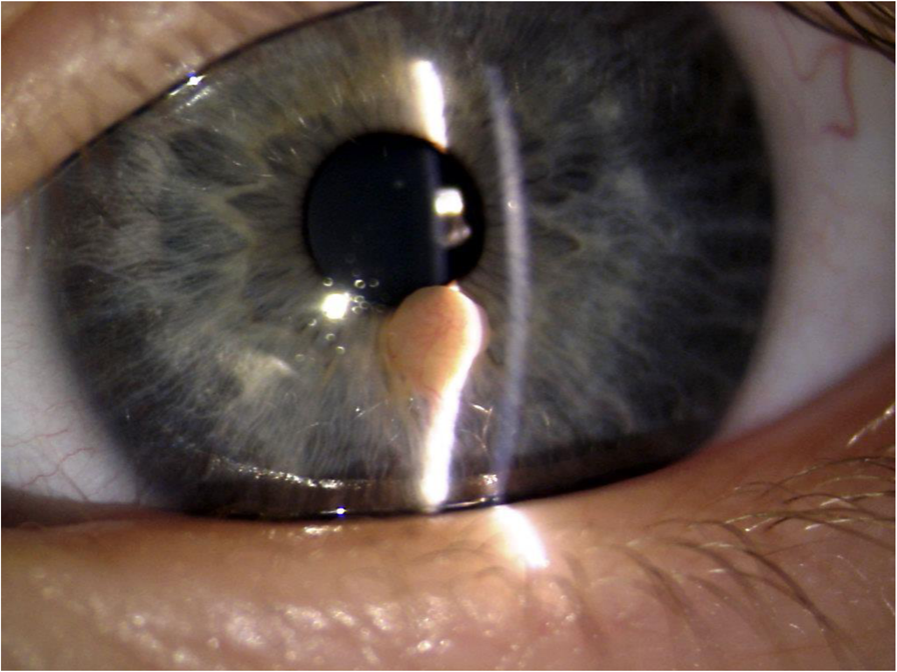
anterior segment slit lamp photography on February 2015 : the picture demonstrates the stability of dimension and vascularization throughout months

The ultrasound biomicroscopy examination showed a well-defined neoformation localized in the iris stroma, not occupying the iris corneal angle, not involving the ciliary body and with low reflectivity. The surrounding iris stroma was thin. (Figs. 2, 3 and 4)

Solid tumors of the iris include melanocytic and nonmelanocytic lesions.

Melanocytic iris tumors comprise a broad spectrum of lesions ranging from benign nevi to aggressive malignant melanomas. Morphologic heterogeneity among melanocytic proliferations is a common challenge in the diagnosis of melanoma [1–3].

The nonmelanocityc lesions are relatively uncommon and include different categories, such as: lacrimal gland choristomas of the iris [4], hemangiomas, neurofibromas, leiomyomas, adenomas and adenocarcinomas [5], xanthomatous lesions, metastatic, lymphoid and leukemic lesions and non-neoplastic mimickers, such us iris cysts or inflammatory lesions [2, 3].

Considering the age of the patient, the growth of the lesion and the morphologic and ultrasound characteristics we were oriented towards small amelanotic iris melanoma, mesoectodermal leyomioma, choristoma or xantoma/xantogranuloma.

A retrospective multicentral study in ophthalmic oncology, demonstrated that most iris melanomas are located in the inferior iris (79.2 %); they are tipically unifocal (88 %). Tumor color is predominantly brown (65 %), followed by amelanotic (9.9 %) and multicolored (6.9 %). Intrinsic tumor vessels are present in 56 % of cases [6]. We found many of these features in this case, which imposed a strict follow up of the patient.

Mesectodermal leiomyoma is a rare tumor originating from smooth muscle, having both muscular and neural differentiation. The first case was reported in 1977, and so far 24 cases have been reported. It should be considered in the differential diagnosis of an amelanotic melanoma, especially in young people [3].

Choristoma is a non-neoplastic, mass-forming lesion containing ectopic or heterologous elements. Although choristomas most commonly involve the epibulbar area, they can affect many areas of the eye and orbit, and often affect more than one area. Choristomas may be associated with coloboma, Goldenhar syndrome or epidermal nevus syndromes; those associated with the latter are often bilateral and extensive. Choristomas are occasionally familial [7].

Most xanthoma/xanthogranuloma of the iris occurs in children. Approximately one half of patients with ocular involvement have skin lesions [2].

The patient underwent follow-up visits every 6 months for two years and was followed annually thereafter.

Tumor stability was assessed by serial UBM and slit lamp photos examinations.

Several features were assessed with the UBM examination: 1) lesion thickness, 2) internal structure (regular and irregular) and 3) internal reflectivity.

A substantial growth was defined if the iris lesion showed an increase in basal diameter and/or thickness (assessed by UBM) of ≥20 % from the previous visit (6 months earlier), or if there is progressive increase in a basal diameter or thickness over 2 or more consecutive visits [8, 9].

The absence of hyphema, seeding of the lesion and intraocular pressure were monitored.

During the 50- month follow up, the lesion did not increase in size, invade iris stroma, nor involve the angle structures. We shared photographs and clinical information with Lausanne Clinic experts in iris tumors, who confirmed our decision to observe.

**Fig. 5:**
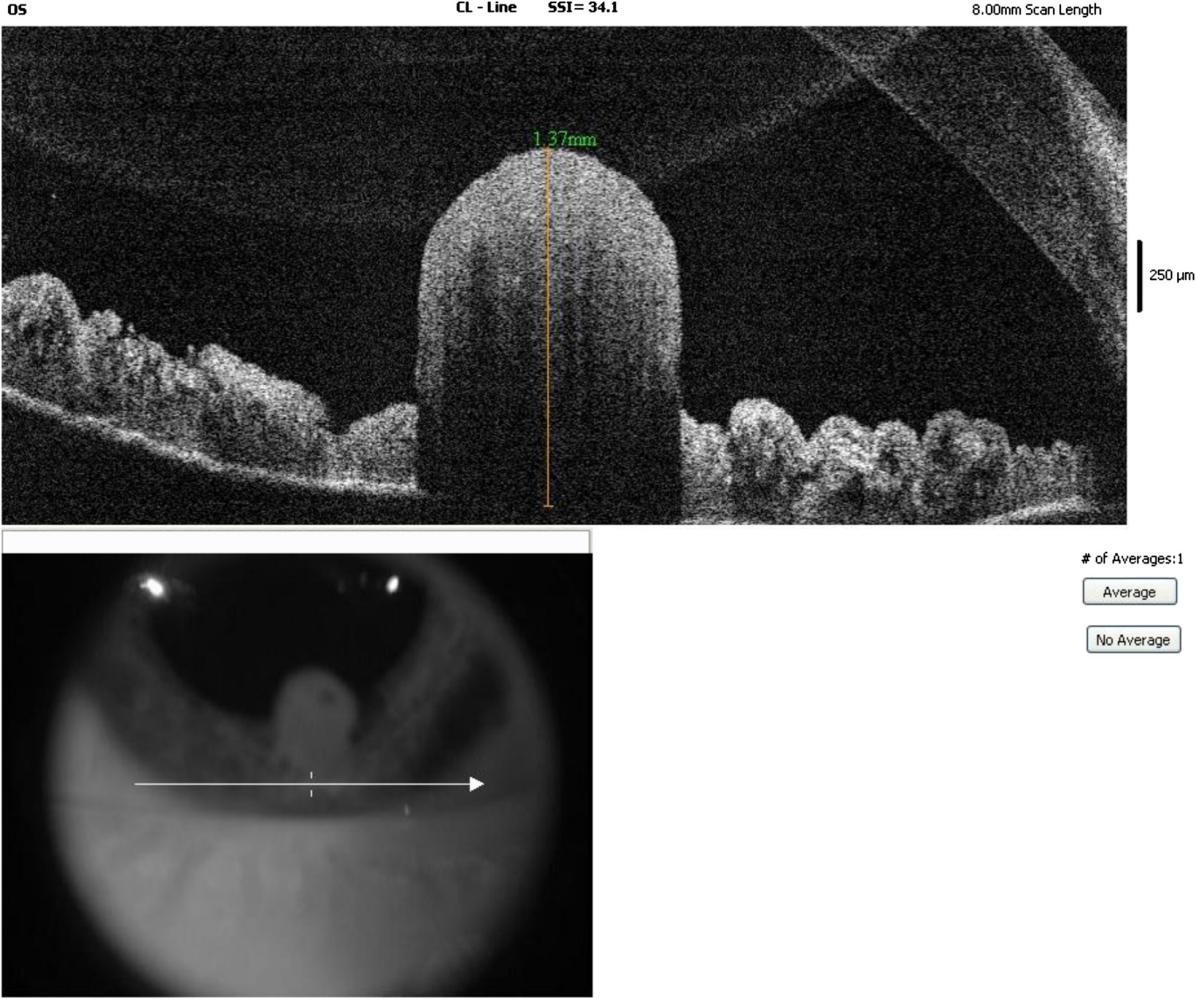
Anterior Segment -OCT (optical coherence tomography) of the lesion on February 2015: the lesion appears well demarcated, not invading the iris stroma nor the ciliary body

During the last visit in February 2015, AS OCT and Scheimpflug camera Image were acquired. (Fig. 5)

## Conclusions

Most of iris tumors in children are cystic, but iris solid neoformations represent a real challenge for the ophthalmologist especially in younger patients. Obtaining reliable images can be challenging, the number of cases is small even in tertiary centers and the presentation is polymorphous and variable. Furthermore even benign melanocytic lesions tend to have a higher rate of growth into melanoma, especially if they involve the inferior quadrants [10].

The case we present has some characteristics of a malignant lesion: it presented with an abrupt growth documented by the parents’ photographs, has an inferior location, is nodular, solitary and vascularized. The size (1,9 mm), the absence of iris infiltration, pupillary distortion, ectropion uveae or glaucoma were suggestive of a benign lesion.

The choice was to proceed with sequential observation of the lesion without proceeding to more invasive procedures because most iris tumors can be diagnosed using clinical and historical criteria without the need for cytologic or pathologic verification [3, 4]. Rarely is a clinical biopsy necessary to confirm the diagnosis of uveal melanoma. The Collaborative Ocular Melanoma Study reported >99 % diagnostic accuracy for eyes with typical features that were enucleated [11].

Fine-needle aspiration biopsy (FNAB) should be reserved for iris tumors in which a diagnosis cannot be obtained and further observation of the lesion could be dangerous to the patient, the eye, or the visual acuity. In addition, some iris tumors are better served with local resection rather than FNAB, depending on tumor location and size, tumor friability, feeder or intrinsic vessels, and the risk of potential seeding of tumor onto the iris surface or anterior chamber [12, 13].

Photographic and UBM documentation of the clinical features can be of great help in diagnosis and follow up of solid iris tumors. It is extremely important to have high quality images in order to document the surface characteristics of the tumor. Serial photographs can also be important to document presence of hyphema, significant vascularization or changes in the pigmentation of the lesion. Tumor size, boundaries, thickness and changes in the internal structure can be followed up by UBM. We did not document the lesion with OCT because at the time of diagnosis, the child was too young to reliably undergo the examination. We recently acquired AS-OCT images because we believe that this less invasive technique can help to further follow the patient, although for anterior segment tumors, UBM offers better visualization of the posterior margin and provides overall better images for entire tumor configuration [14, 15].

Although we were not able to establish a clear-cut diagnosis of the lesion, our approach helped us to exclude a potentially sight threatening lesion. Further follow-up is warranted in such a case, although the timing of the visits may be determined according to the evolution of the lesion. We planned annual follow up visit from now on.

## Abbreviations

AS-OCT, anterior segment optical coherence tomography; FNAB, fine needle aspiration biopsy; UBM, ultrasound biomicroscopy
